# *Borrelia miyamotoi* and *Borrelia burgdorferi* sensu lato widespread in urban areas of the Czech Republic

**DOI:** 10.1186/s13071-024-06549-2

**Published:** 2024-12-18

**Authors:** Alena Balážová, Tomáš Václavík, Vojtech Baláž, Pavel Široký

**Affiliations:** 1 Department of Ecology and Diseases of Zoo Animals, Game, Fish and Bees, University of Veterinary Sciences Brno, Brno, Czech Republic; 2 Department of Ecology and Environmental Sciences, Faculty of Science, Palacký University Olomouc, Šlechtitelů 27, 78371 Olomouc, Czech Republic; 3 Department of Geography, Korea University, 145 Anam-Ro, Seongbuk-Gu, Seoul, 02841 Republic of Korea; 4 Department of Zoology, Faculty of Science, Palacký University Olomouc, Šlechtitelů 27, 78371 Olomouc, Czech Republic; 5 Department of Biology and Wildlife Diseases, Faculty of Veterinary Hygiene and Ecology, University of Veterinary Sciences Brno, Palackého tř. 1946/1, 61242 Brno, Czech Republic; 6 CEITEC-Central European Institute of Technology, University of Veterinary Sciences Brno, Brno, Czech Republic

**Keywords:** *Ixodes ricinus*, Relapsing fever, Minimum infection rate, Estimated prevalence, Geostatistical analysis, Ordinary kriging, Risk maps

## Abstract

**Background:**

*Borrelia miyamotoi* and *Borrelia burgdorferi* sensu lato (s.l.) are important zoonotic agents transmitted by *Ixodes ricinus* ticks, which are widely distributed across Central Europe. Understanding the spatial distribution of these pathogens’ prevalence will help identify areas with increased infection risk and facilitate the implementation of effective preventive measures.

**Methods:**

We analysed 12,955 *I. ricinus* ticks collected from 142 towns in the Czech Republic between 2016 and 2018. The ticks were pooled into 2591 groups of five and tested using duplex quantitative polymerase chain reaction (qPCR) for the presence of *B. burgdorferi* s.l. and *B. miyamotoi*. For each location, we estimated the overall prevalence of both agents using the EpiTools Epidemiological Calculator for pooled samples and calculated the minimum infection rate (MIR). To assess the potential risk of infection, we combined data on the abundance of nymphs and females with pathogen prevalence at each sampled site. Using a geographic information system (GIS), we mapped the MIR and infection risk of both *Borrelia* species across all 142 sampled locations and employed a geostatistical method (ordinary kriging) to predict MIR values and infection risk as continuous surfaces across the entire country.

**Results:**

We detected *B. miyamotoi* in 110 localities and *B. burgdorferi* s.l. in all 142 localities. The estimated prevalence of *B.* *miyamotoi* and *B. burgdorferi* s.l. in the collected ticks was 2.1% (95% confidence interval [CI] 1.8–2.3) and 27.1% (95% CI 26.0–28.3), respectively. For *B. miyamotoi*, we identified previously unknown, geographically distinct hotspots of MIR up to 8.3%, with MIR slightly higher in females (2.3%) than in males (1.9%) and nymphs (1.8%), though the difference was not statistically significant. In contrast, *B. burgdorferi* s.l. exhibited ubiquitous presence, with consistently high prevalence nationwide, showing similar MIRs in females (16.2%) and males (16.1%), and slightly lower in nymphs (15.6%). The highest infection risk for *B. miyamotoi* was 12.4 infected vectors per hour in southeastern Moravia, while the highest risk for *B. burgdorferi* s.l. reached 78.6 infected vectors per hour in the Bohemian-Moravian Highlands.

**Conclusions:**

*Borrelia miyamotoi* is widespread, forming distinct high-prevalence areas in certain regions. *Borrelia* *burgdorferi* s.l. demonstrates consistently high prevalence across most of the country, except for a few localized areas such as southwestern Czechia. Both pathogens exhibit natural nidality, forming regions with elevated prevalence and infection risk. Long-term time-series data are needed to confirm the spatio-temporal stability of these hotspots.

**Graphical Abstract:**

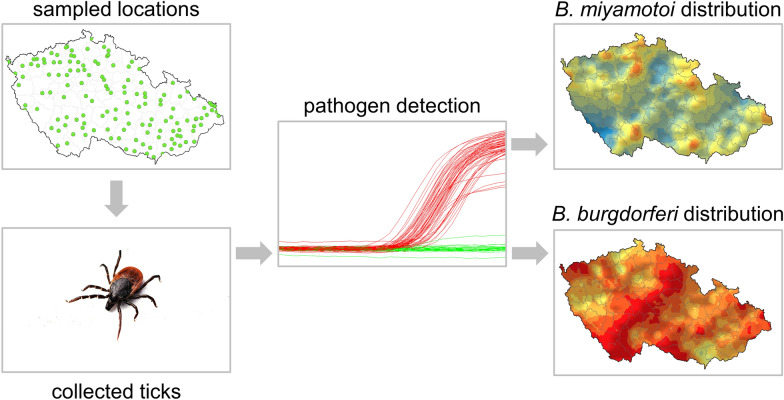

**Supplementary Information:**

The online version contains supplementary material available at 10.1186/s13071-024-06549-2.

## Background

*Borrelia miyamotoi* (Spirochaetaceae, Spirochaetales) belongs to the relapsing fever (RF) group of borreliae. The pathogen is distributed across the entire Holarctic region and forms three distinct geographical lineages: American, European and Asian [1]. It is primarily transmitted by hard ticks, namely *Ixodes persulcatus* (Asia), *Ixodes ricinus* (Europe), *Ixodes scapularis* and *Ixodes pacificus* (America). Unlike Lyme disease borreliae, *B. miyamotoi* exhibits transovarial transmission [2, 3], where larvae hatch already infected, enabling ticks to act as both vectors and reservoirs. The role of other reservoir animals is poorly understood—while the pathogen is occasionally found in various mammals and birds [4], their definitive role as reservoirs and the relative importance of different species remain uncertain. Evidence suggests that infection in rodents may not persist [5]. The zoonotic potential of *B. miyamotoi* has been only recently recognized, exhibiting a disease progression atypical of RF borreliae, with infrequent relapses [6]. Disease symptoms include non-specific manifestations such as fatigue, fever, nausea, myalgia and headaches, with erythema migrans being rare [6]. However, in immunocompromised individuals, the disease can manifest more severely, potentially leading to meningoencephalitis [7].

*Borrelia burgdorferi* sensu lato (s.l.) (Spirochetaceae, Spirochetales) is a complex of 22 genospecies of Gram-negative microaerophilic spirochetes. Ten of these genospecies cause Lyme borreliosis; another 12 were not confirmed to be zoonotic. *Borrelia burgdorferi* s.l. is distributed across the Northern Hemisphere, but the distribution of various genospecies is geographically uneven. The most important genospecies in terms of human Lyme borreliosis are *Borrelia burgdorferi* sensu stricto (s.s.) (America), *Borrelia afzelii* and *Borrelia garinii* (Eurasia). For a better understanding of the genospecies complexity see [8]. Both pathogens have the same vectors. In addition, nine other *Ixodes* species were found capable of transmitting *B. burgdorferi* s.l. [9].

Unlike *B. miyamotoi*, which is known to be transmitted transovarially, it was long believed that *B. burgdorferi* s.l. could not be transmitted in this manner. However, a recent study has challenged this assumption by detecting infection in 0.62% of questing larvae [10]. This finding suggests that some degree of inefficient transmission may be possible. However, the study did not prove that the infection of larvae was transovarial. In either case, the majority of unfed larvae are pathogen-free, with ticks typically acquiring *B. burgdorferi* s.l. infection during their blood meals. This signifies the importance of reservoir hosts for long-term persistence of the pathogen in the environment. Many small mammal species have been recognized as reservoirs, with *B. afzelii* being particularly associated with mammalian hosts [11]. On the other hand, two *Borrelia* species are mostly associated with bird reservoirs—*B. garinii* and *B. valaisiana* [12]. *Borrelia burgdorferi* s.s. can have both mammalian and avian reservoirs [12].

Lyme borreliosis has three stages of progression. The first one is characterized by fever, nausea and erythema migrans occurring in 60–80% of patients [13]. When uncured, the second stage begins in several weeks. The patient is fatigued with myalgia and swollen and painful joints and tendons. The onset of various neurological symptoms is also very common, and other organs can be affected. The third stage starts after months or years of the disease and has severe consequences for the patient—typical symptoms are ataxia, fatigue, inflammation of the skin, heart, brain or eye, and even loss of cognitive functions [13].

Based on the large number of *I. ricinus* ticks collected throughout the Czech Republic as part of a previous project [14], our objective was to investigate the geographical variations in the prevalence of *B. miyamotoi* and *B. burgdorferi* s.l. Furthermore, we sought to analyse the spatial distribution of infection risk levels associated with tick infestations by these pathogenic agents.

## Methods

### Collection of ticks

We used flagging to collect 14,682 *I. ricinus* ticks from 142 towns in the Czech Republic between 2016 and 2018 (Supplementary data). White twill 1 m × 1 m in size was attached to a wooden stick and used as a flag, which was swept through vegetation and along the ground. The sampling sites included mostly woodless meadows, riversides and ecotone habitats separating woodland from grassland (see [14] for details on sampling and site selection). We recorded the date, location coordinates and duration of flagging for each sampled location. All sampled ticks were put into plastic vials, preserved in 96% pure ethanol and transported to the laboratory for species determination. They were then stored at −4 °C until further processing.

### Laboratory sample treatment and PCR diagnosis

The ticks were identified to species level using the key by Nosek and Sixl [15] and pooled in groups of five according to locality, sex and life stage, resulting in 12,955 individuals used for the analysis, grouped in 2591 pools with 735 female, 789 male and 1067 nymph pools. No mixed pools were used. The samples were homogenized using 1 g of 1.4 mm silica beads (BIOplastics, Netherlands) in the MagNA Lyser (Roche Diagnostics) and subsequently isolated using the NucleoSpin^®^ Tissue kit (Macherey-Nagel, Germany) according to manufacturer’s instructions. Detection of *B. burgdorferi* s.l. and *B. miyamotoi* was conducted by a previously published duplex quantitative polymerase chain reaction (qPCR) method [16]. This method uses the same set of primers for both pathogens, which are further distinguished by species-specific probes. In the original study, they used FAM and VIC dyes for their probes; however, we changed VIC for HEX because VIC dye was not available from the manufacturer (EastPort, Praha, Czech Republic). Both VIC and HEX dyes emit light of the same wavelength (580 nm), so the change does not influence the results. The method was tested and optimized before it was routinely used for sample testing. Using thermal-gradient PCR, we confirmed that the ideal annealing and elongation temperature was 63 °C, as published. We tested different primer concentration combinations using a checkerboard method (0.2–0.9 μM) and selected 0.8 μM for both primers, slightly lower than the original study (0.9 μM). Probe concentrations remained at 0.2 μM, as originally suggested. The positive control for *B.* *miyamotoi* was a 10× diluted sample from a bacterial culture (provided by Markéta Nováková, Masaryk University Brno); the positive control for *B. burgdorferi* s.l. was originally also from bacterial culture provided by Masaryk University Brno, and later we used a 10× diluted sample obtained from a rodent host in a previous study [17]. A tested sample was considered positive if the Cp (crossing point value determined by 2nd derivative maximum) was below 40, and showing a standard S-shaped curve.

### Data analysis and GIS modelling

Based on the results of our laboratory analyses, we created maps showing the spatial distribution of both pathogens across the country, following the same approach as in our previous study of overlooked tick-borne pathogens [14]. For all sampled sites, we estimated the overall prevalence of both pathogens using the EpiTools Epidemiological Calculator for pooled samples [18], method 2 (the maximum-likelihood estimator of animal-level prevalence) according to Cowling et al. [19]. As the estimation of true prevalence may be unreliable for small samples, we also used the minimum infection rate (MIR), a conservative measure of infection prevalence, which we calculated as the ratio of the number of positive pools to the total number of individuals in the sample. Using a geographic information system (GIS), we mapped the MIR of *B.* *miyamotoi* and *B. burgdorferi* s.l. at all 142 sampled locations. Additionally, because only female adults and nymphs are likely to infect humans, we assessed the potential risk of infection by combining data on tick abundance and pathogen prevalence for these stages at each sampled location. The risk of infection was calculated as the number of positive female adults or nymphs encountered per hour of flagging [14]. In addition, we employed a geostatistical method to predict the values of MIR and infection risk as continuous surfaces for the entire country. This was done by quantifying the spatial structure of our data with a spatial variogram, fitting a spatial-dependence model to the MIR values, and predicting the values of MIR and infection risk at unsampled locations across the Czech Republic using ordinary kriging [20]. Spatial mapping and analyses were conducted in Geostatistical Analyst, ArcGIS 10.2. (Esri [Environmental Systems Research Institute]).

## Results and discussion

We detected the presence of *B. miyamotoi* in 110 out of 142 (77.5%) localities, with an overall estimated prevalence of 2.1% (95% confidence interval [CI] 1.8–2.3), slightly higher than the Northern Hemisphere prevalence of 1.1% estimated in a previous meta-analysis [21]. Our data align with earlier studies from the Czech Republic reporting prevalence of 0–3.2% [1] and 2.1% [22] and fit within the range of prevalence in vector populations (0.2% to 8.9%) in Europe [23].

**Fig. 1:**
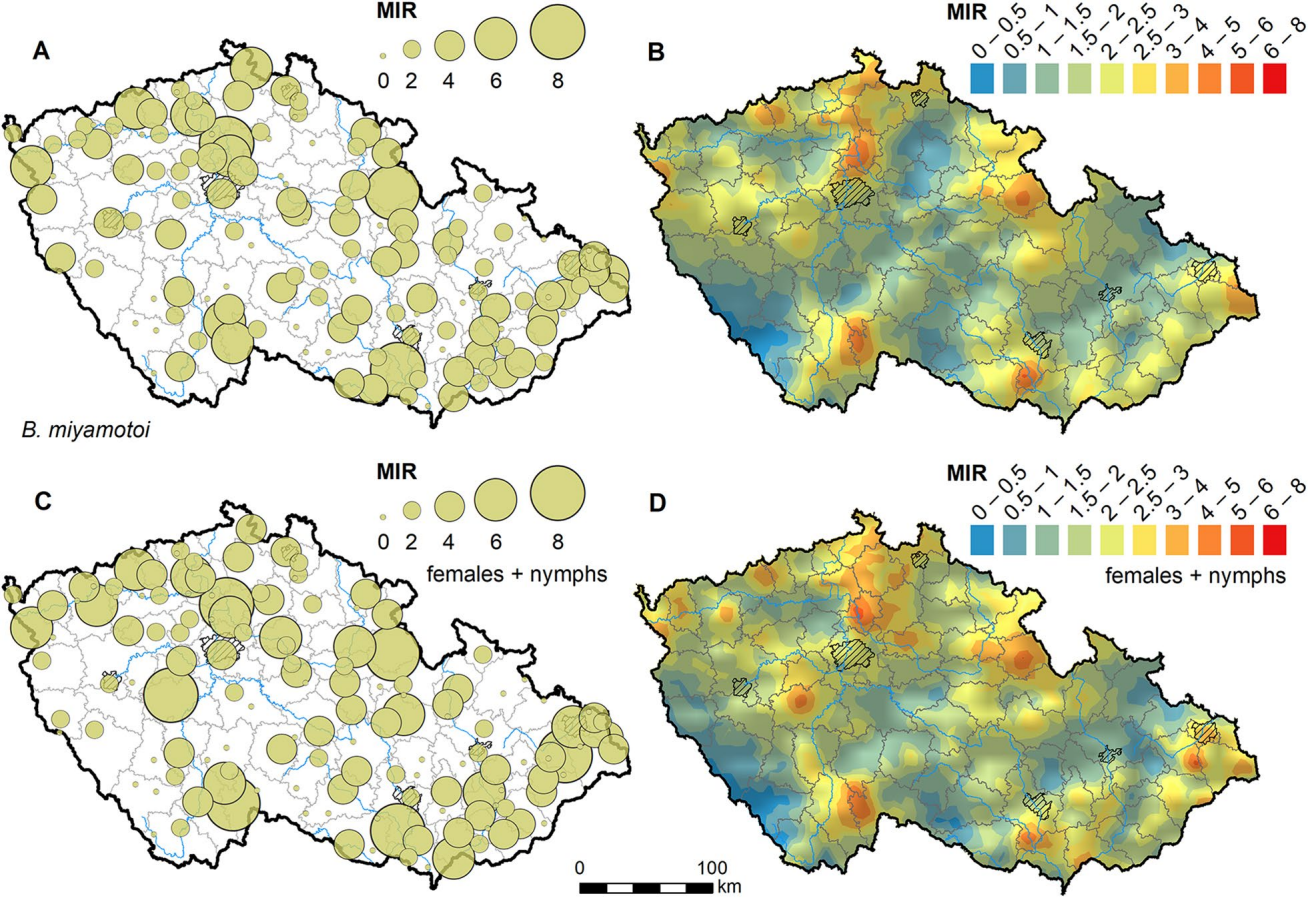
Minimum infection rate (MIR) of *Borrelia miyamotoi* in *Ixodes ricinus* ticks in the Czech Republic. **A** MIR levels measured at sampled locations. **B** MIR levels estimated for the entire country using ordinary kriging. **C** and **D** show MIR levels calculated only for female adults and nymphs (males excluded from the dataset)

The spatial distribution of the pathogen indicates that *B. miyamotoi* is relatively widespread but forms areas of higher prevalence in certain regions (Fig. 1). The MIR was slightly higher in females (2.3%) than males (1.9%) and nymphs (1.8%), though the difference was not statistically significant. Our results also identified previously undetected hotspot areas; the highest MIR of 8.3% was detected at Pohořelice (southeastern Czechia), with similar hotspots found in southwestern and northwestern Czechia. A similar geographical pattern was obtained also after excluding males from the dataset (Fig. 1C and D, Supplementary data). Given the conservative nature of MIR, the actual prevalence of this RF *Borrelia* may be even higher in these natural hotspots.

**Fig. 2:**
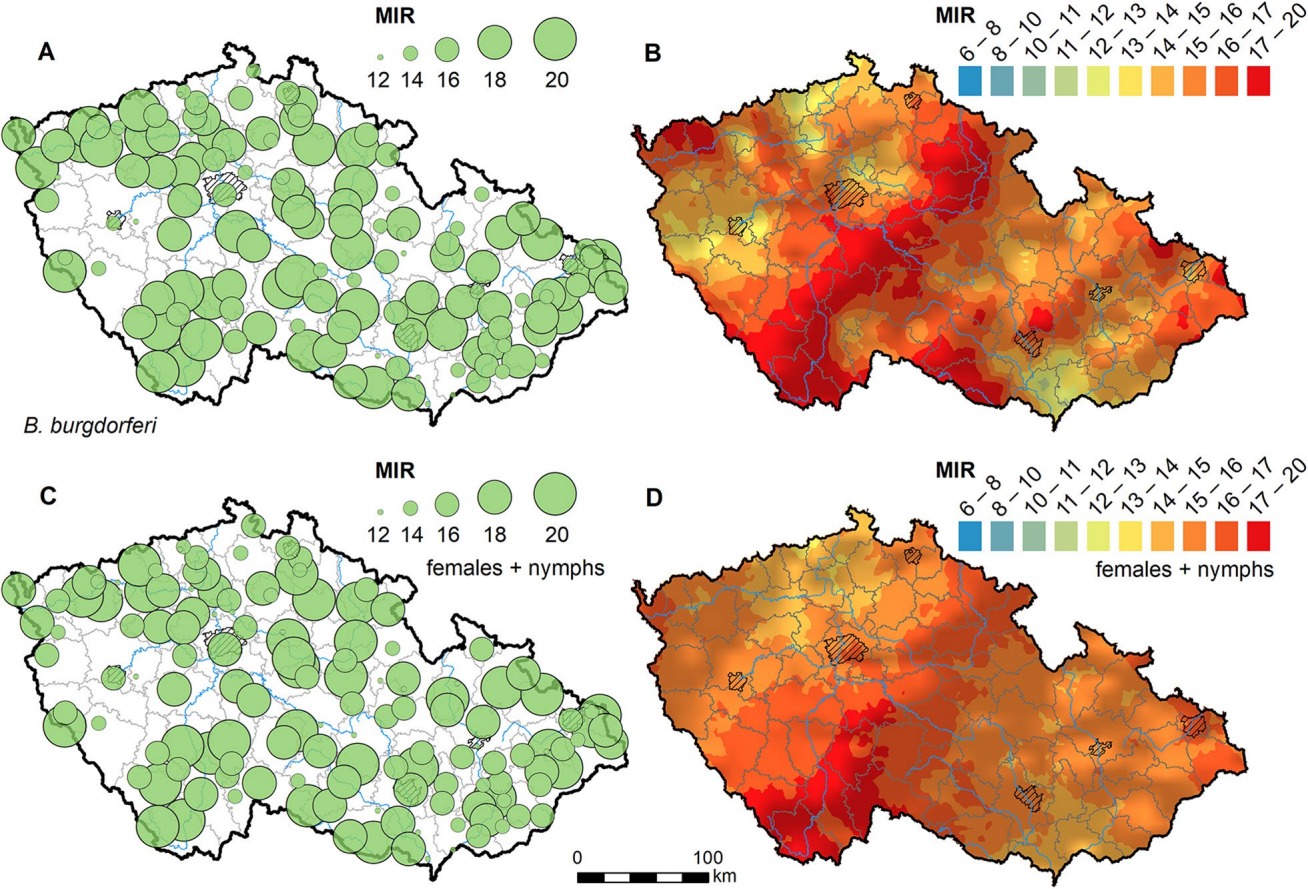
Minimum infection rate (MIR) of *Borrelia burgdorferi* sensu lato in *Ixodes ricinus* ticks in the Czech Republic. **A** MIR levels measured at sampled locations. **B** MIR levels estimated for the entire country using ordinary kriging. **C** and **D** show MIR levels calculated only for female adults and nymphs (males excluded from the dataset)

*Borrelia burgdorferi* s.l. was present at all 142 localities, with an overall estimated prevalence of 27.1% (95% CI 26.0–28.3). The MIR values ranged from 6.7 to 20% (given the method of counting, no MIR can be higher than 20% for our pools), with the maximum MIR observed at seven localities (Fig. 2; Supplementary data). Except for a few smaller areas (e.g. southwestern Czechia), the prevalence of *B.* *burgdorferi* s.l. is consistently high across the whole country. The MIR for *B. burgdorferi* s.l. was similar in females (16.2%) and males (16.1%), and slightly lower in nymphs (15.6%), though the difference was again not statistically significant. Thus, the geographical pattern of MIR was not significantly affected after excluding males from the dataset (Fig. 2C and D). There appears to be an inverse relation in the distribution of *B. burgdorferi* s.l. and *B. miyamotoi* hotspots (Figs. 1 and 2); however, the slightly negative correlation is not statistically significant (*r* = −0.01936164, *P* = 0.8191).

**Fig. 3:**
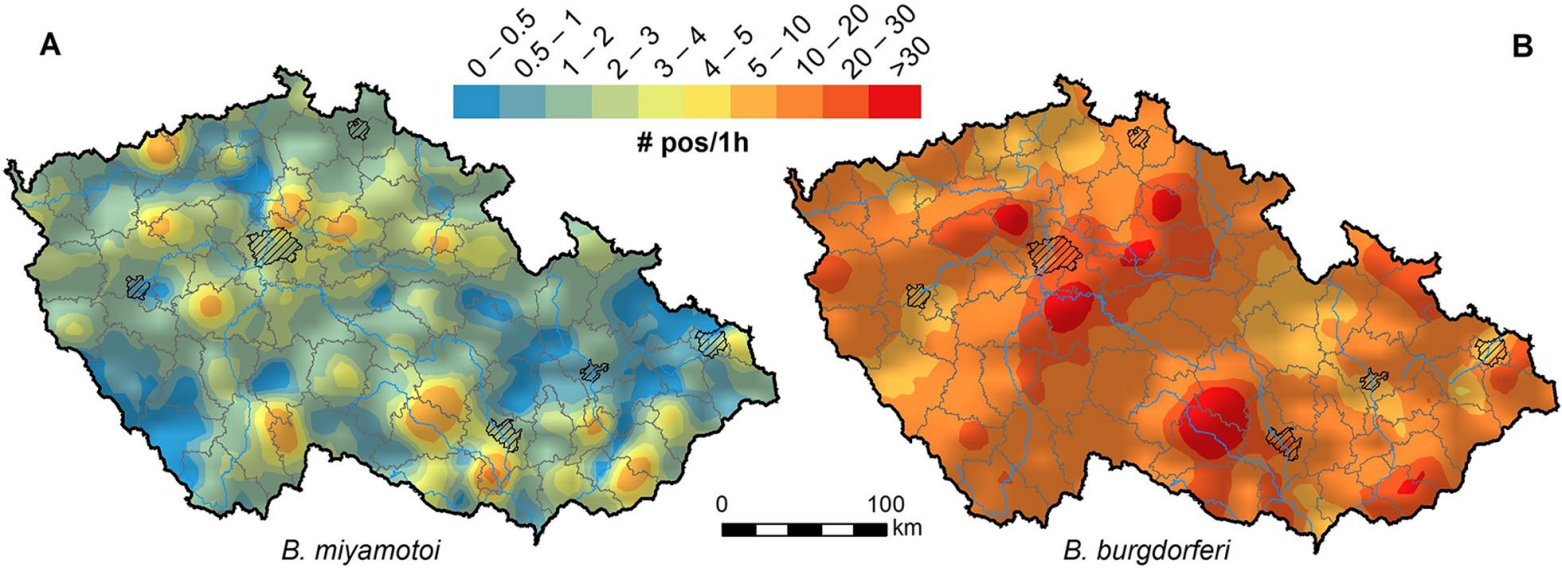
Risk of infection for *Borrelia miyamotoi* (**A**) and *Borrelia burgdorferi* sensu lato (**B**) calculated as the number of positive *Ixodes ricinus* ticks (female adults and nymphs) collected per 1 h of flagging. Continuous risk surfaces created by spatial interpolation (ordinary kriging) of point data

Prevalence alone, especially when based on all collected tick stages, does not directly reflect the risk of disease infection. To provide a clearer measure of infection risk, we present it as the number of positive nymphs and females collected per hour of flagging. A higher number indicates that a host moving through the habitat encounters a greater number of infected nymphs and females, or meets the first infected specimen more quickly. The highest risk of infection by *B. miyamotoi* was detected in Pohořelice (12.4 infected vectors per hour) and Neratovice (11), with three other hotspots also showing more than nine infected nymphs and females per hour (Fig. 3, Supplementary data). For *B. burgdorferi* s.l., we identified the highest risk at Velké Meziříčí, Třebíč and Neratovice (78.6, 71.5 and 69.5 infected ticks per hour, respectively). This means that, on average, a host would encounter the first infected tick within less than 1 min (Fig. 3, Supplementary data).

Our findings align with other studies from the Czech Republic, where the prevalence of *B.* *burgdorferi* s.l. is generally high. Recent studies have reported infection rates of 17.3% [24], 23% [22] and 28.1% [25] in *I. ricinus* ticks. This high prevalence is also reflected in the substantial number of reported human cases every year (3517 cases in 2022 and 3270 in 2023, http://www.szu.cz/publikace/data/2023). Additionally, a seroprevalence study found that 14.8% of sera of healthy patients tested positive for *B. burgdorferi* antibodies [26].

Tick-borne disease agents typically exhibit natural nidality, and the interplay among hosts, vectors and pathogens results in areas with heightened prevalence and infection risk, often showing a level of spatio-temporal stability. However, the limited availability of long-term time-series data has hindered our comprehensive understanding of the dynamics and enduring stability of these hotspots for the studied borreliae. The study presented herein provides a foundation for future observations in this regard. Subsequent research, which would examine epidemiological systems over multiple years and disentangle the combined effect of spatial drivers (e.g. habitat structure) and temporal drivers (e.g. population dynamics of reservoir hosts) affecting tick occurrence and disease prevalence, is needed to confirm or refute the status of these localities as *Borrelia* hotspots and to assess their temporal stability.

## Supplementary Information

**Additional file 1: Dataset S1:** Complete dataset of analysed ticks.

## Data Availability

No datasets were generated or analysed during the current study.

## Abbreviations

MIR: Minimum infection rate
RF: Relapsing fever
GIS: Geographic information system
